# Edible Marine Red Alga *Gracilaria coronopifolia* as a Potential Functional Ingredient: Chemical Profiling and Metabolic Effects in Diet-Induced Obese Rats

**DOI:** 10.3390/foods15071167

**Published:** 2026-03-31

**Authors:** Anton Bahtiar, Larissa Musyantika, Tri Wahyuni, Ratna Annisa Utami, Sirithon Siriamornpun

**Affiliations:** 1 Department of Pharmacology and Toxicology, Faculty of Pharmacy, Universitas Indonesia, Kampus UI Depok, Depok 16424, West Java, Indonesiawahyuni.tri@farmasi.ui.ac.id (T.W.); 2 Laboratory of Pharmaceutical Biotechnology, School of Pharmacy, Institut Teknologi Bandung, Bandung 40132, West Java, Indonesia; ratna.utami@itb.ac.id; 3 Research Unit of Thai Food Innovation, Department of Food Technology, Faculty of Technology, Mahasarakham University, Mahasarakham 44150, Thailand; sirithon.s@msu.ac.th

**Keywords:** marine algae, functional food, diet-induced obesity, polyphenols, lipid metabolism

## Abstract

Marine macroalgae are increasingly recognized as sources of bioactive compounds with potential benefits for metabolic health. This study investigated the chemical composition and metabolic effects of a 70% ethanol extract of the edible red alga *Gracilaria coronopifolia* in a high-fat diet (HFD)-induced obesity model in rats. Chemical profiling using liquid chromatography–high-resolution mass spectrometry (LC–HRMS) identified several classes of metabolites, including sterols, phenolic acids, flavonoids, and fatty acid derivatives such as palmitoleic acid, chlorogenic acid, gallic acid, and quercetin. Male Wistar rats were fed an HFD for 11 weeks to induce obesity and subsequently treated with *G. coronopifolia* extract (40–160 mg/kg body weight) for 28 days, with semaglutide (70 µg/kg) used as a pharmacological comparator. Supplementation with the extract significantly reduced obesity-related parameters compared with untreated HFD controls. The highest extract dose (160 mg/kg) decreased final body weight from **294.8 ± 43.3 g** in HFD rats to **215.2 ± 11.9 g**, reduced visceral fat mass from **22.7 ± 2.37 g** to **7.63 ± 1.19 g**, and lowered the adiposity index from **6.39 ± 0.45%** to **3.31 ± 0.22%**. The extract also improved serum lipid profiles, reducing triglyceride levels from **185.46 ± 11.58 mg/dL** in the HFD group to **101.54 ± 24.29 mg/dL**, while increasing HDL concentrations to **75.64 ± 4.73 mg/dL**. In addition, treatment increased adiponectin levels (to **779.55 ± 15.66**) and decreased leptin (**4.94 ± 0.75**) and amylin (**532.44 ± 30.00**) relative to obese controls. Histological analysis demonstrated a reduction in adipocyte hypertrophy. Gene expression analysis revealed downregulation of hypothalamic *Npy* and adipose *Fas* and *Pparγ*, together with upregulation of *Pomc*, *Mc4r*, and *Cpt1*. These findings suggest that *G. coronopifolia* extract improves metabolic disturbances associated with diet-induced obesity through coordinated regulation of appetite signaling and lipid metabolism, supporting its potential development as a marine-derived functional food ingredient.

## 1. Introduction

Obesity is a chronic and complex disease characterized by excessive fat accumulation that increases the risk of metabolic, cardiovascular, oncological, and reproductive disorders. Its prevalence continues to rise globally, with the number of adults with high body mass index (BMI) projected to increase from 2.2 billion (42% of the adult population) in 2020 to nearly 3.3 billion (54%) by 2035 [1]. This trend is largely driven by modern lifestyle patterns characterized by reduced physical activity and increased consumption of energy-dense, processed foods, resulting in sustained positive energy balance [2].

The primary goal of obesity management is to achieve and maintain weight reduction in order to prevent or ameliorate obesity-related complications and improve quality of life [3]. Lifestyle modification remains the first-line intervention; however, its long-term effectiveness is limited, often necessitating pharmacological support. Glucagon-like peptide-1 receptor (GLP-1R) agonists, such as semaglutide (2.4 mg once weekly in clinical obesity management) have demonstrated clinically meaningful weight loss of up to 15% after 68 weeks of treatment. In the present animal study, semaglutide was included as a pharmacological comparator at 70 µg/kg body weight administered subcutaneously (see Methods) [3]. Despite their efficacy, cost, accessibility, and long-term tolerability remain important considerations, prompting continued interest in alternative or complementary strategies.

Natural products and herbal constituents have gained attention for their potential to modulate GLP-1 secretion and related metabolic pathways [4,5]. Several plant-derived compounds, including berberine, curcumin, quercetin, and resveratrol, have been reported to influence appetite regulation and lipid metabolism, although their weight-loss efficacy generally remains inferior to that of GLP-1R agonists. Consequently, the identification of food-derived bioactive compounds with meaningful metabolic benefits remains a relevant research priority.

Marine macroalgae have gained increasing attention as potential sources of bioactive compounds with beneficial effects on metabolic health [6]. Several studies have reported that extracts from marine algae possess **anti-obesity [7], lipid-lowering [8], antioxidant [9], and anti-inflammatory activities [10]**, which may contribute to the prevention or management of metabolic disorders. Bioactive compounds such as **polyphenols, carotenoids, sulfated polysaccharides, and unsaturated fatty acids** present in marine algae have been shown to modulate lipid metabolism, reduce adipogenesis, and improve metabolic homeostasis in experimental models [11]. In particular, species belonging to the genus *Gracilaria* have been reported to contain phenolic compounds and other metabolites capable of influencing adipocyte differentiation, oxidative stress, and lipid metabolism pathways [12]. These findings highlight the potential of marine algae extracts as promising candidates for the development of functional foods targeting obesity and metabolic syndrome.

Marine macroalgae have attracted increasing attention as potential functional food ingredients due to their rich composition of bioactive compounds, including polyphenols, sterols, carotenoids, and polyunsaturated fatty acids [13]. Among these, **red algae (Rhodophyta)** represent an important group of edible marine organisms widely consumed in various regions and recognized for their diverse biological activities [14]. Several studies have reported that red algae and their extracts may exert beneficial metabolic effects, including antioxidant, anti-inflammatory, and lipid-lowering activities, which are relevant for the prevention and management of obesity-related metabolic disorders [15]. These effects are often attributed to the presence of phenolic compounds, marine lipids, and other secondary metabolites capable of modulating lipid metabolism, adipocyte differentiation, and oxidative stress pathways [16].

Species of the genus ***Gracilaria*** are widely cultivated and consumed edible red algae that have been investigated for their nutritional and pharmacological properties. Previous studies have reported that extracts from certain *Gracilaria* species contain polyphenols and other bioactive metabolites with antioxidant and metabolic regulatory effects [17]. However, despite the growing interest in marine algae as functional food ingredients, **the metabolic effects of** ***G. coronopifolia*** **in the context of diet-induced obesity have not been systematically investigated**.

Therefore, the present study aimed to evaluate the metabolic effects of a **70% ethanol extract of the edible marine red alga** ***G. coronopifolia*** in a **high-fat diet–induced obesity model in rats**. The study investigated its effects on **body weight gain, adiposity parameters, serum lipid profile, adipokine levels, adipocyte morphology, and the expression of genes related to appetite regulation and lipid metabolism**.

Intervention periods of approximately **3–4 weeks are commonly used in rodent models of diet-induced obesity** to evaluate the metabolic effects of dietary bioactive compounds or pharmacological agents [18], as this duration is sufficient to detect changes in body weight regulation, lipid metabolism, and adipokine signaling pathways. Therefore, a **28-day supplementation period** was selected in the present study to evaluate the metabolic effects of *G. coronopifolia* extract following obesity induction.

To date, the metabolic effects of *G. coronopifolia* extract in the context of obesity have not been investigated. Therefore, the present study aimed to evaluate the effects of supplementation with a 70% ethanol extract of *G. coronopifolia* on obesity-associated metabolic alterations in a high-fat diet–induced obese rat model. The study assessed anthropometric parameters, adiposity indices, lipid profiles, adipokine levels, adipocyte morphology, and the expression of genes involved in appetite regulation and lipid metabolism, with semaglutide included as a pharmacological comparator.

## 2. Materials and Methods

### 2.1. Materials

Fresh red algae (*G. coronopifolia*) (5 kg, fresh weight) were collected from the cultivation facility of the Research Institute for Medicinal and Aromatic Plants (Balai Penelitian Tanaman Obat dan Aromatik, Balitro), Ministry of Agriculture, Cimanggu, Bogor, Indonesia, in March 2025. The species was taxonomically authenticated, and a voucher specimen No. J125-P-51 was deposited at the Herbarium Depokensis of Universitas Indonesia.

### 2.2. Preparation of G. coronopifolia Red Algae Extract

Fresh *G. coronopifolia* samples were thoroughly washed with water and oven-dried at **55–60 °C for 24 h until a constant weight was achieved**. The final dried material had a **moisture content of approximately 8.6% (*****w*****/*****w*****)**, indicating effective dehydration prior to extraction. The dried material was then ground into a fine powder using a blender before extraction. Extraction was performed by maceration in 70% ethanol at a sample-to-solvent ratio of 1:3 (*w*/*v*) at room temperature with intermittent stirring [19]. The mixture was allowed to stand for 24 h, and the maceration process was repeated three times. Combined filtrates were filtered through Whatman filter paper and concentrated under reduced pressure using a rotary vacuum evaporator at 40 °C to obtain a viscous crude extract [20].

### 2.3. Identification of Compounds in G. coronopifolia Red Algae Extract

The chemical constituents of the concentrated *G. coronopifolia* extract were analyzed at the Corpora Science Research Laboratory using Liquid Chromatography–High Resolution Mass Spectrometry (LC-HRMS). Chromatographic separation was performed on a Thermo Scientific™ Vanquish™ Horizon UHPLC system equipped (Thermo Fisher Scientific, Waltham, MA, USA) with a binary pump and an Accucore™ Phenyl-Hexyl analytical column (100 × 2.1 mm, 2.6 µm particle size, Thermo Fisher Scientific, Waltham, MA, USA). The column temperature was maintained at 40 °C, with a flow rate of 0.3 mL/min and an injection volume of 5 µL [21].

The mobile phase consisted of (A) MS-grade water containing 0.1% formic acid and (B) MS-grade acetonitrile containing 0.1% formic acid. The gradient program started at 5% B, increased linearly to 90% B over 16 min, held at 90% B for 4 min, and then returned to 5% B, with a total run time of 25 min.

Mass spectrometric detection was carried out using a Thermo Scientific™ Orbitrap™ Exploris 240 high-resolution mass spectrometer equipped with an OptaMax™ NG heated electrospray ionization (H-ESI) source. Data were acquired in full MS/dd-MS^2^ mode with polarity switching. Full MS scans were collected at a resolution of 60,000 FWHM over an *m*/*z* range of 70–800, with a maximum injection time of 100 ms and a mass tolerance of 5 ppm. Data-dependent MS^2^ scans were acquired at a resolution of 30,000 FWHM using normalized collision energies of 30, 50, and 70, with nitrogen as the collision gas [21].

Ion source parameters were set as follows: spray voltage 3500 V (positive) and 2500 V (negative), sheath gas 35 AU, auxiliary gas 7 AU, sweep gas 1 AU, ion transfer tube temperature 300 °C, and vaporizer temperature 320 °C.

For metabolomic sample preparation, 20 mg of extract was dissolved in 1 mL of HPLC-grade methanol, vortex-mixed for 2 min, sonicated for 30 min, and centrifuged at 1400× *g* for 5 min. The supernatant was filtered through a 0.20 µm nylon membrane filter before injection.

Data processing and compound annotation were performed using Thermo Scientific™ Compound Discoverer™ software version 3.3. Tentative compound identification was based on accurate mass measurements, MS/MS fragmentation patterns, and comparison with multiple databases, including mzCloud, ChemSpider, KEGG, LIPID MAPS, Natural Products Atlas, and flavonoid and endogenous metabolite mass lists.

The chemical composition of the *G. coronopifolia* extract was characterized using LC–HRMS analysis. The extract contained multiple classes of metabolites, including sterols, fatty acids, phenolic acids, flavonoids, and amide derivatives. Among the tentatively identified compounds were 22-dehydrocholesterol, palmitoleic acid, chlorogenic acid, gallic acid, p-coumaric acid, quercetin, and several polyunsaturated fatty acid derivatives. These metabolites were identified based on accurate mass measurements and MS/MS spectral matching against public databases. The extract was dominated by sterol-related compounds, with **22-dehydrocholesterol representing the most abundant identified metabolite** based on relative peak area.

### 2.4. Preparation of Semaglutide Injection Solution and G. coronopifolia Red Algae Extract Solution

Semaglutide injection was prepared aseptically in a laminar air flow cabinet using sterile equipment [22]. A sterile vehicle solution consisting of 44 mM Na_2_HPO_4_, 70 mM NaCl, and 0.007% Tween 20 in water for injection (WFI) was prepared and sterilized by sequential filtration through 0.45 µm and 0.22 µm membrane filters. Sterile semaglutide was aseptically dissolved in the vehicle, aliquoted into individual vials, and stored at 2–8 °C until use for subcutaneous administration over a 28-day treatment period.

The *G. coronopifolia* extract was dissolved in the same vehicle immediately prior to oral administration and was not sterilized. Although semaglutide was administered subcutaneously, treatment groups are collectively referred to as ‘dietary supplementation groups’ for comparative purposes

### 2.5. High-Fat-Diet Animal Model

Male Wistar rats (7 weeks old; 120–140 g) were obtained from Kemuning, an accredited laboratory animal supplier in Central Java, Indonesia. Following an acclimatization period, rats assigned to the obesity model were fed a high-fat diet (HFD) from day 15 to day 91 of the study. Obesity was confirmed based on a body weight gain greater than 50%, a Lee index exceeding 315, and a body mass index (BMI) greater than 0.68, in accordance with established criteria [23].

Male Wistar rats were used in this study to reduce variability related to the estrous cycle, which can significantly influence body weight regulation, adiposity, lipid metabolism, and adipokine profiles, thereby allowing clearer assessment of the metabolic effects of dietary supplementation [24].

### 2.6. High-Fat Diet (HFD) Composition

The high-fat diet (HFD) used for obesity induction was prepared by modifying standard laboratory chow with additional fat and carbohydrate sources. The diet consisted of normal chow (50%), beef tallow (20%), sucrose (20%), and butter (10%) (*w*/*w*) [25]. All components were thoroughly mixed to obtain a homogeneous formulation and were provided daily during the obesity induction period.

The normal control group received standard laboratory chow, while animals in the HFD groups were fed the formulated high-fat diet throughout the induction phase.

### 2.7. Dietary Supplementation of Test Animals

Body weight and body length were measured regularly throughout the study. Animals were housed under standard laboratory conditions with ad libitum access to food and water, replenished daily between 08:00 and 10:00 h [26]. Baseline blood samples were obtained before initiation of the high-fat diet (HFD).

After the obesity induction period, rats were allocated into experimental groups and received the assigned dietary supplementation for 28 days. The negative control group consisted of high-fat diet (HFD)-fed rats receiving no treatment, while the positive control group consisted of HFD-fed rats treated with semaglutide (70 µg/kg body weight, subcutaneously), a GLP-1 receptor agonist used as a pharmacological reference for anti-obesity effects. Rats in the treatment groups received *G. coronopifolia* extract at doses of 40, 80, or 160 mg/kg body weight, while the normal control group was maintained on standard chow. Blood samples were collected at the end of the dietary supplementation period for subsequent biochemical, hormonal, and molecular analyses, including lipid profiling, adipokine measurement, and gene expression assessment.

### 2.8. Food Intake Measurement

Food intake was monitored daily throughout the experimental period. Each rat was housed individually in a separate cage to allow accurate measurement of individual food consumption. The amount of food provided to each rat was weighed at the beginning of the light cycle, and the remaining food was weighed after 24 h. Daily food intake was calculated as the difference between the amount of food provided and the remaining food. Average daily food intake was expressed as grams per rat per day.

### 2.9. Lee Index and Body Mass Index

The Lee index and body mass index (BMI) were determined weekly to evaluate obesity status. Body weight was measured using an analytical balance, and naso-anal length was measured from the nose to the anus using a flexible string and recorded to the nearest 0.1 cm. The Lee index was calculated using the formula:

$$\mathrm{Lee}\;\mathrm{index}=\frac{\sqrt[{3}]{\mathrm{body}\;\mathrm{weight}\;(g)}}{\mathrm{naso-anal}\;\mathrm{length}\;(\mathrm{cm})}\times 10^{3}$$

Rats with a Lee index > 315 were classified as obese. BMI was calculated as body weight (g) divided by the square of naso-anal length (cm^2^), with values > 0.68 indicating obesity.

### 2.10. Adiposity Index and Visceral Fat Weight

After the dietary supplementation period, rats were anesthetized with ketamine hydrochloride (100 mg/kg) and xylazine (5 mg/kg) administered intraperitoneally, followed by euthanasia. A thoracotomy was performed, and visceral adipose tissues, including epididymal, perirenal, and retroperitoneal fat pads, were excised and weighed. Total visceral fat mass was calculated as the sum of these depots.

The adiposity index was calculated using the following formula:

Adiposity index (%) = (total visceral fat weight/final body weight) × 100.

### 2.11. Histological Analysis of Adipose Tissue (Hematoxylin and Eosin Staining)

Epididymal adipose tissue samples were collected immediately after euthanasia and fixed in **10% neutral-buffered formalin for 24 h**. The fixed tissues were then dehydrated through a graded ethanol series, cleared in xylene, and embedded in paraffin wax.

Paraffin blocks were sectioned into **5 µm-thick slices** using a rotary microtome and mounted on glass slides. Tissue sections were deparaffinized in xylene, rehydrated through decreasing concentrations of ethanol, and rinsed in distilled water.

Sections were stained with **hematoxylin for 5 min**, rinsed in running tap water, differentiated in acid alcohol, and blued in alkaline solution. Subsequently, the sections were counterstained with **eosin for 1–2 min**, dehydrated through graded ethanol, cleared in xylene, and mounted with coverslips using a permanent mounting medium.

Stained sections were observed using a **light microscope**, and digital images were captured for morphological evaluation. Adipocyte diameter and circumference were measured using image analysis software to quantify adipocyte hypertrophy.

### 2.12. Lipid Profile

Serum lipid profiles were analyzed using blood samples collected from overnight-fasted rats. Rats were anesthetized with ketamine hydrochloride (75 mg/kg, intraperitoneal), and blood was collected from the retro-orbital sinus into Eppendorf tubes. Blood samples were allowed to clot and then centrifuged to obtain serum for biochemical analysis.

#### 2.12.1. Triglycerides

Triglyceride levels were measured by mixing 10 µL of serum with 1000 µL of triglyceride reagent. The mixture was incubated for 20 min at 20–25 °C, and absorbance was measured spectrophotometrically at 500 nm, according to the manufacturer’s instructions.

#### 2.12.2. HDL

HDL cholesterol was determined following precipitation of non-HDL lipoproteins. Briefly, 200 µL of serum was mixed with 500 µL of precipitation reagent and centrifuged at 2000–2500× *g* for 20 min. A volume of 100 µL of the resulting supernatant was mixed with 1000 µL of cholesterol reagent and incubated at 37 °C for 10 min. Absorbance was measured at 500 nm.

#### 2.12.3. Total Cholesterol

Total cholesterol levels were measured by mixing 10 µL of serum with 1000 µL of cholesterol reagent, followed by incubation for 20 min at 20–25 °C. Absorbance was read spectrophotometrically at 500 nm.

### 2.13. Serum Adipokine Levels

Serum leptin, amylin, and adiponectin concentrations were measured using commercially available enzyme-linked immunosorbent assay kits according to the manufacturers’ instructions (ELISA). Rat leptin, adiponectin, and amylin ELISA kits were obtained from [Fine Test, Wuhan, China] (Leptin: Cat. No. ER0115; Adiponectin: Cat. No. ER0006; Amylin: Cat. No. ER0374).

Blood samples were collected from overnight-fasted rats, allowed to clot, and centrifuged at 3000× *g* for 15 min at 4 °C to obtain serum. Samples were stored at −80 °C and thawed only once before analysis. All samples were analyzed in duplicate.

The intra-assay coefficients of variation (CVs) were <4.68% for leptin, <4.68% for adiponectin, and <5.29% for amylin, while inter-assay CVs were <4.89%, <5.23%, and <5.37%, respectively, as reported by the manufacturer. Absorbance was measured using a microplate reader, and concentrations were calculated from standard curves generated for each assay.

### 2.14. Quantitative Real-Time PCR Analysis of Appetite- and Lipid Metabolism–Related Genes

Quantitative real-time PCR (qRT-PCR) was performed to evaluate the expression of appetite-regulating genes **Neuropeptide Y (Npy), Pro-opiomelanocortin (Pomc), and Melanocortin 4 Receptor (Mc4r)** in hypothalamic tissue and lipid metabolism–related genes Peroxisome Proliferator-Activated Receptor gamma *(Pparγ)*, Adiponectin Receptor 1 *(Adipor1)*, *Fas*, and *Cpt-1* in peripheral adipose tissue. **β-Actin was used as the housekeeping gene for normalization of gene expression in both hypothalamic and adipose tissues.** β-Actin was selected as the internal reference gene because of its stable expression across experimental conditions. Relative gene expression levels were calculated using the **2^−ΔΔCt^ method**.

After completion of the dietary supplementation period, rats were euthanized, and the hypothalamus and visceral adipose tissues were rapidly excised, snap-frozen in liquid nitrogen, and stored at −80 °C until analysis. Total RNA was extracted from each tissue using the TRIzol™ RNA Mini Kit (Qiagen, Germantown, MD, USA), according to the manufacturer’s instructions. RNA concentration and purity were assessed spectrophotometrically, and complementary DNA (cDNA) was synthesized using the SuperScript™ III Reverse Transcription Kit.

Gene expression of *Fas* and *Cpt-1* was analyzed using fluorescence-based quantitative PCR diagnostic kits. Amplification reactions were performed using a Rotor-Gene Q 5plex real-time PCR system. For *Fas*, the PCR protocol consisted of an initial incubation at 50 °C for 5 min, followed by initial denaturation at 95 °C for 40 s and 34 cycles of amplification at 95 °C for 40 s, 59 °C for 40 s, and 72 °C for 40 s, with a final extension at 72 °C for 2 min. Fluorescence signals were recorded at the end of each cycle, and β-actin was used as the housekeeping gene.

For *Cpt-1*, the PCR protocol included an initial incubation at 37 °C for 5 min, initial denaturation at 95 °C for 5 min, and 40 amplification cycles consisting of 95 °C for 10 s, 60 °C for 20 s, and 70 °C for 1 s. Target gene concentrations were calculated based on standard curves generated using positive reference controls provided in the kits.

Expression levels of hypothalamic *Npy*, *Pomc*, and *Mc4r*, as well as adipose *Pparγ* and *Adipor1*, were quantified using qRT-PCR under optimized cycling conditions, normalized to appropriate housekeeping genes, and expressed as relative fold changes using the 2^−ΔΔCt^ method [27,28,29].

Based on the outcomes of anthropometric, biochemical, and histological evaluations, only the highest-dose level group of *G. coronopifolia* extract (GCE3; 160 mg/kg body weight) was selected for molecular analysis. This group demonstrated the most consistent and significant improvements across measured obesity-related parameters; therefore, it was prioritized for gene expression assessment to evaluate potential molecular responses associated with the observed physiological effects.

### 2.15. Statistical Analysis

All data are expressed as **mean ± standard deviation (SD)** for each experimental group (*n* = 6). Statistical analysis was performed using **one-way analysis of variance (ANOVA)** to evaluate differences among groups, followed by **Tukey’s post hoc multiple comparison test** when appropriate. Statistical significance among groups is indicated by different superscript letters in the tables; groups that do not share the same letter differ significantly (*p* < 0.05).

## 3. Results

### 3.1. Chemical Characterization of G. coronopifolia Extract

Untargeted LC–HRMS analysis was performed to characterize the chemical composition of the 70% ethanol extract of *G. coronopifolia*. The total ion chromatogram (TIC) obtained from the analysis is presented in Figure S1 (Supplementary Materials), showing multiple chromatographic peaks across the retention time window, indicating the presence of diverse metabolites within the extract.

Based on accurate mass measurements and MS/MS spectral matching, several metabolites belonging to different chemical classes were tentatively identified. These included sterols, fatty acid derivatives, phenolic acids, flavonoids, and amide-type compounds [30]. The identified metabolites and their corresponding characteristics are summarized in Table 1.

Among the identified metabolites, sterol-related compounds represented a notable component of the extract, with **22-dehydrocholesterol** showing the highest relative peak area. Fatty acid–related metabolites included palmitoleic acid and polyunsaturated fatty acid derivatives such as dihomogamma-linolenic acid ethyl ester and (+/−)18-HEPE. Additionally, several phenolic compounds were detected, including **chlorogenic acid, gallic acid, and p-coumaric acid**, along with the flavonoid **quercetin**. The presence of these metabolites suggests that the extract contains bioactive constituents potentially capable of modulating metabolic processes as previously described [31].

### 3.2. Effects of G. coronopifolia Extract on Obesity-Related Physiological Parameters

High-fat diet (HFD) induction was performed in male Wistar rats following a 14-day acclimatization period. Animals were fed either standard chow (normal control) or an HFD composed of normal chow, beef tallow, butter, and sucrose [32,33]. After 11 weeks, HFD-fed rats met established obesity criteria, including >50% body weight gain, a Lee index > 315, and a body mass index (BMI) >0.68 [34].

Following confirmation of obesity, dietary supplementation was initiated. Rats were allocated into six groups: normal control (non-HFD), HFD control, HFD + semaglutide (SEMA), and three HFD groups receiving *G. coronopifolia* extract at 40 (GCE1), 80 (GCE2), or 160 mg/kg body weight (GCE3). The effects of dietary supplementation on body weight gain, Lee index, and BMI are summarized in Table 2.

Table 2 summarizes the effects of supplementation with *G. coronopifolia* extract on body weight gain, Lee index, and body mass index (BMI) in high-fat diet (HFD)-fed rats. HFD feeding resulted in significant increases in all three parameters compared with the normal control group (*p* < 0.05), confirming successful obesity induction.

Following the 28-day supplementation period, treatment with semaglutide and *G. coronopifolia* extract resulted in improvements in these anthropometric parameters compared with the untreated HFD group. The effects were more pronounced at higher extract doses, particularly in the **GCE3 group (160 mg/kg body weight)**.

#### 3.2.1. Weight Gain

**Figure 1:**
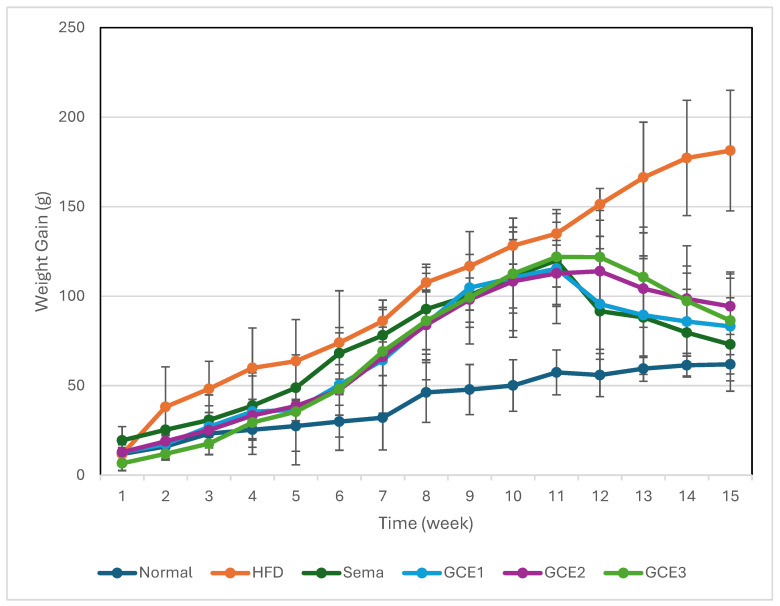
Effects of supplementation with *G. coronopifolia* extract on body weight gain in high-fat diet (HFD)-fed rats over 15 weeks. HFD: high-fat diet; Sema: Semaglutide, 70 µg/kg BW; GCE1–GCE3: *G. coronopifolia* extract (40, 80, and 160 mg/kg body weight). Values are expressed as mean ± SD (*n* = 6).

Changes in body weight during the experimental period are illustrated in Figure 1. Rats fed the HFD exhibited a marked increase in body weight compared with the normal control group. In contrast, treatment with semaglutide and *G. coronopifolia* extract reduced body weight gain relative to the untreated HFD group.

Among the extract-treated groups, the greatest reduction in body weight gain was observed in the **GCE3 group**, suggesting a dose-dependent effect of the extract on weight regulation.

#### 3.2.2. Lee Index and Body Mass Index (BMI)

The Lee index was used as an additional indicator of obesity status. HFD feeding significantly increased the Lee index compared with the normal control group, reflecting increased adiposity. Following treatment, both semaglutide and *G. coronopifolia* extract reduced the Lee index relative to the HFD group, with the most pronounced reduction observed in the highest-dose extract group.

Similarly, BMI values increased significantly in HFD-fed rats compared with normal controls. Treatment with semaglutide and the extract reduced BMI values, with the largest decrease observed in the **GCE3 group**, indicating improvement in obesity-related anthropometric parameters.

#### 3.2.3. The Effects of G. coronopifolia Extract on Food Intake, Adiposity-Related Parameters, and Adipocyte Morphology in High-Fat Diet (HFD)-Induced Obese Rats

Daily food intake was monitored throughout the experimental period to assess potential changes in feeding behavior. During the HFD induction period, food intake among groups was relatively comparable, ranging from 13.6 to 15.5 g/day, indicating similar baseline feeding behavior. After the treatment period, rats in the semaglutide group exhibited the lowest food intake (9.2 ± 2.3 g/day), followed by the highest extract dose group (GCE3; 9.4 ± 1.0 g/day), suggesting that the extract may influence appetite regulation. The average daily food intake for each experimental group is summarized in Table 3.

HFD feeding markedly increased visceral fat accumulation and adiposity index compared with the normal group. Total visceral fat increased from 4.02 ± 0.79 g in the normal group to 22.7 ± 2.37 g in HFD rats, while the adiposity index increased from 1.88 ± 0.27% to 6.39 ± 0.45%. Treatment with semaglutide and *G. coronopifolia* extract significantly reduced these parameters. In particular, the highest extract dose (GCE3) reduced visceral fat mass to 7.63 ± 1.19 g and the adiposity index to 3.31 ± 0.22%, approaching the values observed in the semaglutide-treated group.

Histological measurements also demonstrated that HFD feeding induced marked adipocyte hypertrophy. Adipocyte circumference increased from 179.99 ± 21.66 µm in the normal group to 301.56 ± 25.09 µm in HFD rats, while adipocyte diameter increased from 57.29 ± 6.89 µm to 95.99 ± 7.99 µm. Treatment with semaglutide and *G. coronopifolia* extract reduced adipocyte size, with the greatest improvement observed in the GCE3 group (236.10 ± 31.84 µm circumference; 75.15 ± 10.13 µm diameter).

Overall, these findings indicate that *G. coronopifolia* extract attenuated HFD-induced obesity by reducing food intake, decreasing visceral fat accumulation, and limiting adipocyte hypertrophy, with the strongest effects observed at the highest extract dose.

**Figure 2:**
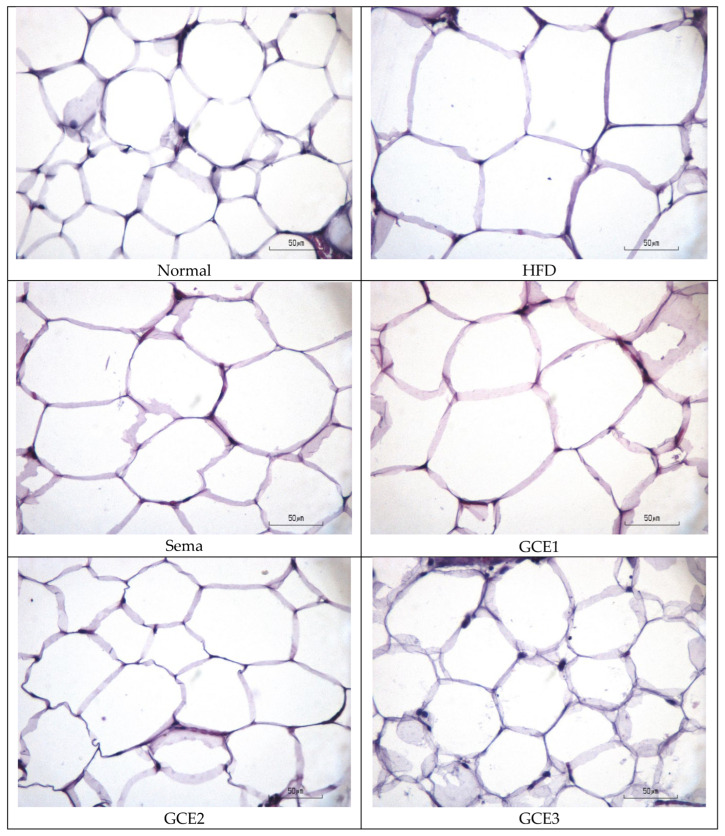
Representative hematoxylin and eosin–stained sections of epididymal adipose tissue from (Normal) normal control, (HFD) HFD control, (SEMA) HFD + Semaglutide, 70 µg/kg BW, (GCE1) HFD + *G. coronopifolia* extract 40 mg/kgBW, (GCE2) 80 mg/kgBW, and (highest dose (GCE3)) 160 mg/kgBW groups. Supplementation with *G. coronopifolia* extracts attenuated adipocyte hypertrophy compared with HFD control. Scale bar = 50 µm.

Quantitative measurements of adipocyte circumference and diameter are summarized in Table 3, while representative histological images are shown in Figure 2.

### 3.3. Effects of G. coronopifolia Extract on Metabolic Biomarkers and Gene Expression

#### 3.3.1. Effects of *G. coronopifolia* Extract on Serum Adipokines and Lipid Profile in High-Fat Diet (HFD)-Induced Obese Rats

Serum adipokine concentrations were evaluated to determine the metabolic effects of the treatments. HFD feeding significantly reduced adiponectin levels and increased leptin and amylin concentrations compared with the normal control group, indicating metabolic dysregulation associated with obesity, as shown in Table 4.

HFD feeding markedly disrupted metabolic biomarkers compared with the normal control group. In particular, adiponectin levels decreased substantially from **831.47 ± 16.55** in the normal group to **176.01 ± 12.86** in the HFD group, while leptin and amylin concentrations increased to **9.25 ± 0.52** and **1006.93 ± 28.09**, respectively, indicating obesity-associated endocrine dysregulation.

Treatment with semaglutide and *G. coronopifolia* extract improved these alterations in adipokines. The highest extract dose (GCE3) markedly increased adiponectin levels to **779.55 ± 15.66**, approaching the values observed in the semaglutide-treated group (**736.86 ± 23.05**). In parallel, leptin and amylin concentrations were reduced in extract-treated groups, with GCE3 decreasing leptin to **4.94 ± 0.75** and amylin to **532.44 ± 30.00**, suggesting partial normalization of adipose tissue endocrine function.

HFD feeding also significantly worsened lipid parameters, increasing total cholesterol from **81.52 ± 4.94 mg/dL** in normal rats to **125.17 ± 1.77 mg/dL**, and triglycerides from **92.23 ± 5.54 mg/dL** to **185.46 ± 11.58 mg/dL**, while reducing HDL levels from **130.04 ± 20.90 mg/dL** to **59.71 ± 4.63 mg/dL**. Supplementation with *G. coronopifolia* extract improved these lipid abnormalities. The highest extract dose reduced triglyceride levels to **101.54 ± 24.29 mg/dL**, which was comparable to the levels in the semaglutide-treated group (115.13 ± 6.48 mg/dL). Total cholesterol levels decreased to **83.39 ± 3.68 mg/dL**. HDL concentrations were also significantly increased in extract-treated groups relative to the HFD group.

Overall, these results indicate that *G. coronopifolia* extract effectively ameliorated obesity-associated metabolic disturbances by improving adipokine balance and restoring serum lipid profiles in HFD-fed rats.

#### 3.3.2. Gene Expression Analysis Related to Appetite Regulation and Lipid Metabolism

To explore potential molecular mechanisms underlying the observed metabolic effects, quantitative real-time PCR analysis was performed to evaluate the expression of genes related to appetite regulation and lipid metabolism. HFD feeding significantly increased hypothalamic expression of ***Npy*** while decreasing ***Pomc*** and ***Mc4r***, indicating dysregulation of appetite control pathways. Supplementation with *G. coronopifolia* extract reversed these changes, particularly in the highest-dose group (GCE3), suggesting restoration of normal hypothalamic signaling.

In adipose tissue, HFD feeding increased the expression of the adipogenic transcription factor ***Pparγ*** and the lipogenic enzyme ***Fas***, while reducing ***Cpt1***, a key enzyme involved in mitochondrial fatty acid oxidation. Treatment with the extract reduced ***Pparγ*** and ***Fas*** expression while increasing ***Cpt1***, indicating a shift toward enhanced lipid oxidation and reduced lipogenesis. Gene expression data are presented in Table 5.

Quantitative real-time PCR analysis was performed to assess the effects of *G. coronopifolia* extract on genes involved in appetite regulation and lipid metabolism, as shown in Table 5. Gene expression levels were calculated using the 2^−ΔΔCt^ method and are expressed relative to the normal control group.

High-fat diet (HFD) feeding significantly altered hypothalamic expression of appetite-related genes, with upregulation of the orexigenic gene *Npy* and downregulation of the anorexigenic genes *Pomc* and *Mc4r* compared with the normal control group. Supplementation with *G. coronopifolia* extract resulted in the most pronounced effects observed at the highest dose normalization of these changes, with the highest dose (GCE3, 160 mg/kg) producing the most pronounced effects.

HFD feeding also significantly affected adipose tissue expression of genes related to lipid metabolism. Expression of the adipogenic transcription factor *Pparγ* and the lipogenic enzyme *Fas* was increased, while expression of *Cpt1* was reduced. Supplementation with *G. coronopifolia* extract significantly downregulated *Pparγ* and *Fas* expression and upregulated *Cpt1*, particularly at the highest dose. Expression of *Adipor1* was also restored toward control levels in extract-treated groups.

## 4. Discussion

### 4.1. Chemical Characterization and Biological Relevance

LC–HRMS profiling revealed that *G. coronopifolia* extract contains diverse bioactive metabolites, including sterols, fatty acid derivatives, phenolic acids, and flavonoids. Major compounds included 22-dehydrocholesterol (2.04%), p-coumaric acid (0.07%), palmitoleic acid (0.07%), chlorogenic acid (0.03%), gallic acid (0.03%), and quercetin (0.02%). The predominance of sterol-related compounds is consistent with marine macroalgae, where sterols typically account for approximately 1–5% of detected metabolites [8].

Although phenolic compounds were present at relatively low levels (<0.1% per compound), their biological activity remains significant. Previous studies have shown that quercetin and chlorogenic acid can reduce triglyceride levels by 25–30% and body weight by 10–15% in HFD-fed rodents [35,36]. Similarly, marine-derived unsaturated fatty acids have been reported to decrease triglycerides by 20–40% through activation of PPAR signaling pathways [37]. In addition, marine sterols can reduce circulating cholesterol by approximately 10–20% [38].

Taken together, these findings suggest that the metabolite profile of *G. coronopifolia*, although quantitatively modest at the individual compound level, is functionally comparable to other bioactive-rich plant and algae extracts. The coexistence of polyphenols, marine lipids, and sterols likely enables synergistic interactions, contributing to their metabolic regulatory potential.

### 4.2. Physiological and Morphological Outcomes

Supplementation with *G. coronopifolia* extract significantly improved obesity-related physiological parameters, including body weight, adiposity, and food intake. At the highest dose (160 mg/kg), body weight was reduced by approximately 27% compared with HFD controls (294.8 g to 215.2 g), exceeding reductions reported for common natural compounds such as green tea polyphenols and resveratrol (10–20%) [39,40].

Visceral fat mass decreased by approximately 66% (22.7 g to 7.63 g), which is greater than reductions typically reported for plant- and algae-derived extracts (20–50%) [41]. This pronounced reduction in adiposity indicates a strong effect of *G. coronopifolia* on fat accumulation and storage.

The extract also significantly reduced food intake by approximately 45% (17.2 to 9.4 g/day), exceeding the appetite suppression commonly reported for polyphenols (15–30%) [42]. This reduction was accompanied by modulation of hypothalamic appetite-regulating genes, including downregulation of *Npy* and upregulation of *Pomc* and *Mc4r* [43], indicating restoration of energy homeostasis.

Histological analysis further demonstrated attenuation of adipocyte hypertrophy, with reduced adipocyte size consistent with decreased visceral fat mass. Similar morphological improvements have been reported for marine algae extracts [44], although the magnitude observed in this study suggests a comparatively stronger effect.

Overall, these findings indicate that *G. coronopifolia* exerts anti-obesity effects through combined regulation of energy intake, adiposity, and adipose tissue morphology.

### 4.3. Metabolic Biomarkers and Molecular Mechanisms

The extract also significantly improved metabolic biomarkers, including adipokines and lipid profiles. Adiponectin levels increased approximately 4.4-fold (176.01 to 779.55), exceeding the 1.5–3-fold increases typically reported for polyphenol-rich interventions [45,46]. In parallel, leptin levels decreased by approximately 47%, indicating improved leptin sensitivity and reduced adiposity.

Serum triglyceride levels were reduced by approximately 45% (185.46 to 101.54 mg/dL), which is greater than reductions commonly reported for quercetin or curcumin (20–35%) [47]. HDL concentrations also increased substantially, reaching values at the upper range reported for marine algae interventions.

At the molecular level, *G. coronopifolia* extract modulated key genes involved in appetite regulation and lipid metabolism. The normalization of hypothalamic *Npy*, *Pomc*, and *Mc4r* expression was consistent with the observed ~45% reduction in food intake, supporting a central mechanism of appetite control [48]. Similar but less pronounced effects have been reported for polyphenols and marine lipids.

In adipose tissue, downregulation of *Fas* and *Pparγ*, along with upregulation of *Cpt1*, indicates suppression of lipogenesis and enhancement of fatty acid oxidation. These molecular changes are consistent with the observed improvements in lipid profiles and adiposity and align with previous findings for marine-derived bioactive compounds [49].

Collectively, these results demonstrate that *G. coronopifolia* exerts anti-obesity effects through coordinated modulation of central appetite regulation and peripheral lipid metabolism, with effects that are quantitatively comparable to or greater than those reported for established natural compounds.

### 4.4. Positioning Novelty

Importantly, while several marine algae species have demonstrated anti-obesity potential, **no previous quantitative data are available for** ***G. coronopifolia***. Therefore, the present findings not only confirm its metabolic benefits but also suggest that its efficacy is comparable to, or in some parameters exceeds, that of previously studied plant- and algae-derived bioactive compounds.

## 5. Conclusions

Supplementation with *Gracilaria coronopifolia* ethanol extract significantly ameliorated obesity-associated metabolic disturbances in a high-fat diet–induced obese rat model. The extract, particularly at 160 mg/kg body weight, reduced body weight by approximately **27%**, visceral fat mass by **~66%**, and triglyceride levels by **~45%**, while markedly increasing adiponectin levels (~4.4-fold) and improving HDL concentrations. These effects were accompanied by reduced food intake (~45%), attenuation of adipocyte hypertrophy, and favorable modulation of genes involved in appetite regulation (*Npy*, *Pomc*, *Mc4r*) and lipid metabolism (*Fas*, *Pparγ*, *Cpt1*).

Importantly, the magnitude of these effects is comparable to or exceeds those reported for several plant-derived polyphenols and marine bioactive compounds, highlighting the strong metabolic efficacy of *G. coronopifolia*. The observed benefits are likely attributable to the combined and potentially synergistic actions of polyphenols, marine lipids, and sterol-related metabolites identified in the extract.

To the best of our knowledge, this study provides the first comprehensive evidence of the anti-obesity effects of *G. coronopifolia*, demonstrating its ability to modulate both central appetite regulation and peripheral lipid metabolism. These findings support its potential development as a marine-derived functional food ingredient for the prevention and management of metabolic disorders.

Nevertheless, as a preclinical study, further research is required to validate these effects in long-term studies and clinical settings, as well as to elucidate the contribution of individual bioactive compounds and their mechanisms of action.

### Study Limitations

Several limitations of the present study should be acknowledged. First, phytochemical characterization of the *G. coronopifolia* extract was qualitative, and the concentrations of individual bioactive compounds were not quantified. Consequently, the specific contribution of individual constituents to the observed metabolic effects could not be determined. Future studies should include compound isolation and dose–response analyses of individual metabolites.

Second, only male rats were used in this study to minimize variability associated with hormonal fluctuations. Therefore, sex-specific differences in response to *G. coronopifolia* extract cannot be excluded, and future studies should include female animals to evaluate potential sex-dependent effects.

Third, although significant changes in gene expression related to appetite regulation and lipid metabolism were observed, protein-level validation and functional assays were not performed. As mRNA expression does not necessarily correlate with protein abundance or enzymatic activity, these molecular findings should be interpreted as indicative rather than definitive. Future investigations should incorporate protein-based analyses, such as Western blotting, immunohistochemistry, or enzyme activity assays, to confirm the functional relevance of the transcriptional changes.

Finally, classical inflammatory markers associated with obesity-related low-grade inflammation, such as interleukin-6 and C-reactive protein, were not assessed. While inclusion of these biomarkers would have strengthened mechanistic interpretation, this represents a moderate limitation and does not detract from the observed metabolic and hormonal improvements. Future studies should incorporate inflammatory cytokine profiling to further clarify the role of inflammation in the metabolic effects of *G. coronopifolia* extract.

## Figures and Tables

**Table 1:** Tentatively identified metabolites in the 70% ethanol extract of *G. coronopifolia* based on LC-HRMS analysis.

RT (min)	*m*/*z*	Formula	Tentative Identification	Class	Relative Peak Area (%)	MSI Level
14.022	367.336	C27H44O	22-dehydrocholesterol	Sterol	2.0387	Level 2
1.065	182.081	C9H8O3	p-coumaric acid	Phenolic acid	0.0749	Level 2
13.108	255.232	C16H30O2	Palmitoleic acid	Monounsaturated fatty acid (MUFA)	0.0652	Level 2
16.203	584.488	C35H61N5O2	N-[(2S)-1-{4-[{4[(3,3-dimethylbutyl)amino] phenyl}(3methylbutyl)amino]piperidin-1-yl}-4-methyl-1-oxopentan-2-yl]azepane-1-carboxamide	Amide	0.0505	Level 2
0.941	311.259	C19H36O3	10-oxo-nonadecanoic acid	Oxo-fatty acid (fatty acid derivative)	0.0487	Level 2
2.715	353.088	C16H18O9	Chlorogenic acid	Phenolic acid	0.0321	Level 2
1.206	169.014	C7H6O5	Gallic acid	Phenolic acid	0.0308	Level 2
13.713	335.294	C22H38O2	Dihomo-γ-linolenic acid ethyl ester	PUFA (ω-6)	0.0269	Level 2
4.694	303.05	C15H10O7	Quercetin	Flavonoid	0.0203	Level 2
10.859	317.212	C20H30O3	(+/−)18-HEPE	PUFA derivative (ω-3 oxylipin)	0.0127	Level 2

**Note:** Metabolite annotations were assigned according to the Metabolomics Standards Initiative (MSI) criteria. Identification Level 2 indicates putative identification based on accurate mass and MS/MS spectral matching against public databases. Authentic reference standards were not used to confirm retention times or molecular structures.

**Table 2:** Effects of *G. coronopifolia* extract on anthropometric parameters in high-fat diet-fed rats after 28 days of supplementation.

Group	Final Body Weight (g)	Lee Index	BMI
Normal	197.1 ± 13.7	321.9 ± 11.6	0.60 ± 0.0
HFD	294.8 ± 43.3 ^a^	377.5 ± 21.9 ^a^	0.95 ± 0.2 ^a^
Sema	221.7 ± 36.1	345.6 ± 27.6	0.73 ± 0.1 ^b^
GCE1	213.1 ± 27.8 ^b^	336.9 ± 20.3	0.68 ± 0.1 ^b^
GCE2	220.7 ± 32.8	342.1 ± 16.7	0.71 ± 0.1 ^b^
GCE3	215.2 ± 11.9 ^b^	326.1 ± 8.6 ^b^	0.64 ± 0.1 ^b^

Note: HFD: high-fat diet; Sema: Semaglutide, 70 µg/kg BW; GCE1–GCE3: *G. coronopifolia* extract (40, 80, and 160 mg/kg body weight). Values are expressed as mean ± SD (*n* = 6). (^a^) indicate statistically significant differences compared with Normal groups. (^b^) indicate statistically significant differences compared with HFD groups (*p* < 0.05; one-way ANOVA followed by Tukey’s test).

**Table 3:** Effects of *Gracilaria coronopifolia* extract on food intake, adiposity parameters, and adipocyte morphology in high-fat diet (HFD)-induced obese rats after 28 days of treatment.

Parameters		Normal	HFD	Sema	GCE1	GCE2	GCE3
**Food intake**	**HFD induction period (g/day)**	14.4 ± 1.1	15.5 ± 1.8	11.9 ± 0.6	14.2 ± 1.7	14.2 ± 0.8	13.6 ± 1.0
	**After treatment (g/day)**	17.0 ± 1.2	17.2 ± 2.2	9.2 ± 2.3 ^ab^	13.1 ± 1.3 ^ab^	11.5 ± 0.9 ^ab^	9.4 ± 1.0 ^ab^
**Adiposity parameters**	**Total Visceral Fat (g)**	4.02 ± 0.8	22.7 ± 2.3 ^a^	5.45 ± 0.7 ^b^	12.42 ± 1.2 ^b^	9.27 ± 1.06 ^b^	7.63 ± 1.2 ^b^
	**Adiposity index (%)**	1.88 ± 0.3	6.39 ± 0.4 ^a^	2.37 ± 0.48 ^b^	5.51 ± 0.8	4.63 ± 0.4 ^b^	3.31 ± 0.22 ^b^
**Adipocyte morphology**	**Circumference of the cell circle (µm)**	179.99 ± 21.7	301.56 ± 25.1 ^a^	219.71 ± 40.4 ^b^	273.32 ± 22.5 ^a^	261.56 ± 38.7 ^a^	236.10 ± 31.8 ^b^
	**Diameter (µm)**	57.29 ± 6.9	95.99 ± 7.9 ^a^	69.94 ± 12.9 ^b^	87.00 ± 7.2 ^a^	83.26 ± 12.3 ^a^	75.15 ± 10.1 ^b^

**Note:** HFD: high-fat diet; Sema: Semaglutide, 70 µg/kg BW; GCE1–GCE3: *G. coronopifolia* extract (40, 80, and 160 mg/kg body weight). Values are expressed as mean ± SD (*n* = 6). (^a^) indicate statistically significant differences compared with Normal groups. (^b^) indicate statistically significant differences compared with HFD groups (*p* < 0.05; one-way ANOVA followed by Tukey’s test).

**Table 4:** Effects of *G. coronopifolia* extract on serum adipokines and lipid profile in high-fat diet (HFD)-induced obese rats after 28 days of treatment.

		Normal	HFD	Sema	GCE1	GCE2	GCE3
**Serum adipokines**	**Adiponectin**	831.47 ± 16.55	176.01 ± 12.86 ^a^	736.86 ± 23.05 ^b^	478.19 ± 57.02 ^ab^	541.02 ± 37.23 ^ab^	779.55 ± 15.66 ^b^
	**Leptin**	3.79 ± 0.71	9.25 ± 0.52 ^a^	4.52 ± 0.17 ^b^	8.05 ± 0.47 ^a^	6.06 ± 0.67 ^ab^	4.94 ± 0.75 ^ab^
	**Amylin**	458.56 ± 32.49	1006.93 ± 28.09 ^a^	510.04 ± 14.57 ^b^	796.76 ± 37.8 ^ab^	651.56 ± 49.08 ^ab^	532.44 ± 30.00 ^ab^
**Lipid profiles**	**Total cholesterol (mg/dL)**	81.52 ± 4.94	125.17 ± 1.77 ^a^	84.89 ± 7.36 ^b^	85.28 ± 3.64 ^b^	83.22 ± 3.44 ^b^	83.39 ± 3.68 ^b^
	**Triglycerides (mg/dL)**	92.23 ± 5.54	185.46 ± 11.58 ^a^	115.13 ± 6.48 ^b^	143.26 ± 21.75 ^ab^	129.11 ± 33.43 ^b^	101.54 ± 24.29 ^b^
	**HDL (mg/dL)**	130.04 ± 20.90	59.71 ± 4.63 ^a^	79.17 ± 3.63 ^b^	87.08 ± 3.53 ^b^	86.43 ± 4.41 ^b^	75.64 ± 4.73 ^b^

**Note:** HFD: high-fat diet; Sema: Semaglutide, 70 µg/kg BW; GCE1–GCE3: *G. coronopifolia* extract (40, 80, and 160 mg/kg body weight). Values are expressed as mean ± SD (*n* = 6). (^a^) indicate statistically significant differences compared with Normal groups. (^b^) indicate statistically significant differences compared with HFD groups (*p* < 0.05; one-way ANOVA followed by Tukey’s test.

**Table 5:** Effects of dietary supplementation with *G. coronopifolia* extract on gene expression related to appetite regulation and lipid metabolism.

	(A) Hypothalamus			(B) Adipose Tissue			
	*Npy*	*Pomc*	*Mc4r*	*Fas*	*Cpt-1*	*Adipor1*	*Pparγ*
Normal	1	1	1	1	1	1	1
HFD	17.8 ± 1.9 ^a^	0.72 ± 0.04 ^a^	0.57 ± 0.05 ^a^	2.53 ± 0.1 ^a^	n.d	1.63 ± 0.4 ^a^	3.95 ± 0.4 ^a^
Sema	9.3 ± 0.9 ^ab^	1.77 ± 0.15 ^ab^	1.19 ± 0.23 ^b^	3.53 ± 0.2 ^ab^	2.26 ± 0.05 ^a^	1.65 ± 0.6 ^b^	0.82 ± 0.1 ^b^
GCE3	1.22 ± 0.2 ^b^	1.98 ± 0.29 ^ab^	1.36 ± 0.12 ^b^	1.6 ± 0.4 ^ab^	2.48 ± 0.4 ^a^	0.95 ± 0.5 ^b^	2.42 ± 0.7 ^ab^

Note: Only the GCE3 group (160 mg/kg body weight) was included in gene expression analysis because it exhibited the most pronounced effects in anthropometric, biochemical, and histological assessments. HFD: high-fat diet; Sema: Semaglutide, 70 µg/kg BW; GCE3: *G. coronopifolia* extract 160 mg/kg body weight. Values are expressed as mean ± SD (*n* = 6). (^a^) indicate statistically significant differences compared with Normal groups. (^b^) indicate statistically significant differences compared with HFD groups (*p* < 0.05; one-way ANOVA followed by Tukey’s test. n.d: undetectable under assay conditions.

## Data Availability

The original contributions presented in this study are included in the article/Supplementary Material. Further inquiries can be directed to the corresponding author.

## Supplementary Materials

The following supporting information can be downloaded at: https://www.mdpi.com/article/10.3390/foods15071167/s1. Figure S1. Total ion chromatogram (TIC) of the 70% ethanol extract of *Gracilaria coronopifolia* obtained by LC–HRMS analysis.
