# Supplementary material for: Edible Marine Red Alga Gracilaria coronopifolia as a Potential Functional Ingredient: Chemical Profiling and Metabolic Effects in Diet-Induced Obese Rats
Source: Foods. 2026 Mar 31;15(7):1167. doi: 10.3390/foods15071167 (PMC13073850; doi:10.3390/foods15071167)
Supplement: Supplementary file 1 [file foods-15-01167-s001.zip › foods-4182946-supplementary.pdf]

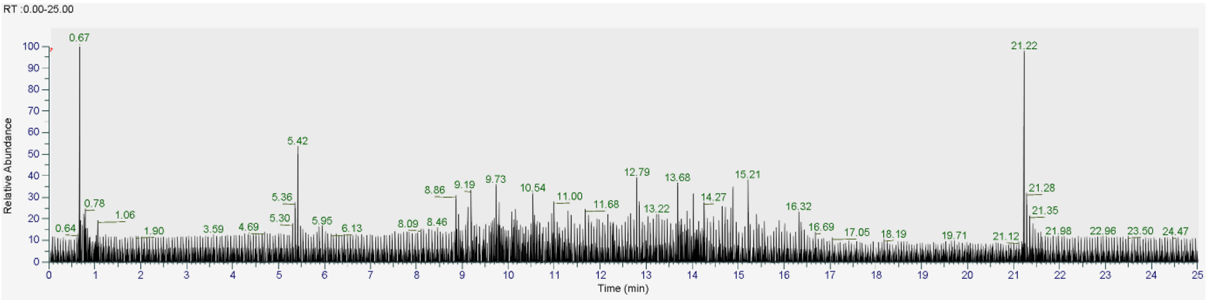

Figure S1. Total ion chromatogram (TIC) of the 70% ethanol extract of *Gracilaria coronopifolia* obtained by LC–HRMS analysis.
